# 3D mesoporous structure assembled from monoclinic M-phase VO_2_ nanoflakes with enhanced thermochromic performance†

**DOI:** 10.1039/d1ra01558c

**Published:** 2021-04-13

**Authors:** Liboro Hundito Molloro, Shouqin Tain, Neway Belachew, Kwadwo Asare Owusu, Xiujian Zhao

**Affiliations:** State Key Laboratory of Silicate Materials for Architectures, Wuhan University of Technology (WUT) No. 122, Luoshi Road Wuhan 430070 P. R. China liborotg@yahoo.com opluse@whut.edu.cn; Department of Chemistry, Debre Berhan University P.O. Box 445 Debre Berhan Ethiopia; State Key Laboratory of Advanced Technology for Materials Synthesis and Processing, Wuhan University of Technology (WUT) No. 122, Luoshi Road Wuhan 430070 P. R. China

## Abstract

Monoclinic M-phase VO_2_ is a promising candidate for thermochromic materials due to its abrupt change in the near infrared (NIR) transmittance along with the metal-to-insulator transition (MIT) at a critical temperature ∼68 °C. However, low luminous transmittance (*T*_lum_), poor solar energy modulation ability (Δ*T*_sol_), and high phase transition temperature (*T*_c_) can limit the application of VO_2_ for smart windows. To overcome these limitations, 3D mesoporous structure can be employed in VO_2_ films. Herein, 3D mesoporous structures assembled from monoclinic M-phase VO_2_ nanoflakes with a pore size of about 2–10 nm were synthesized by a hydrothermal method using *Ensete ventricosum* fiber (EF) as a template followed by calcination at 450 °C. The prepared film exhibited excellent thermochromic performance with balanced *T*_lum_ = 67.3%, Δ*T*_sol_ = 12.5%, and lowering *T*_c_ to 63.15 °C. This is because the 3D mesoporous structure can offer the uniform dispersion of VO_2_ nanoflakes in the film to enhance *T*_lum_, ensure sufficient VO_2_ nanoflakes in the film for high Δ*T*_sol_ and lower *T*_c_. Therefore, this work can provide a green approach to synthesize 3D mesoporous structures assembled from monoclinic M-phase VO_2_ nanoflakes and promote their application in smart windows.

## Abstract 2

Herein, 3D mesoporous structures assembled from monoclinic M-phase VO_2_ nanoflakes were successfully synthesized for enhanced thermochromic performance.

## Graphical abstract

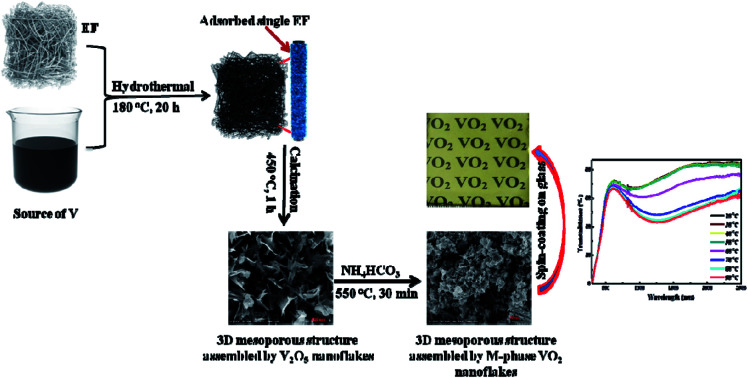

## 1. Introduction

The demand for energy-efficient smart windows utilizing thermochromic materials is constantly increasing due to their potential to dramatically reduce the energy consumption of the buildings by modulating the transmission of light and heat.^3–7^ Vanadium dioxide (VO_2_) has been widely investigated for smart windows because of its excellent thermochromic properties.^8–10^ In particular, monoclinic M-phase VO_2_ undergoes a metal-to-insulator transition (MIT) at a critical temperature of ∼68 °C.^12–14^ The monoclinic crystal structure of VO_2_ displays infrared transparent below the critical temperature while in a rutile crystal structure it is infrared-reflective above the critical temperature accompanied by abrupt changes in the electrical, magnetic, and optical properties.^1,16–18^ Due to this special MIT property, monoclinic M-phase VO_2_ is a promising candidate for the thermochromic smart window.^23,24^ However, low luminous transmittance (*T*_lum_), which is not higher than ∼40%,^16,27^ poor solar energy modulation ability (Δ*T*_sol_), which is usually less than 10%^16,27^ and high phase transition temperature (*T*_c_) ∼68 °C, which should be lowered to room temperature,^1,13,20^ limits the application of VO_2_ in smart windows. Up to the present, various attempts have been made to overcome these limitations,^13,31^ including introducing pores,^29,30,32^ structural modification,^2,35^ designing 2D nanoarchitecture,^7,33,34^ core–shell,^24^ multilayer stack design,^36^ doping,^1,20^ composite^15,22,26^ and film thickness optimization.^37^ Optical calculation confirms that incorporation of VO_2_ in another dielectric material could increase *T*_lum_ and Δ*T*_sol_.^27,38^ The simulation conducted by Taylor *et al.*^39^ and the sample prepared by Qian *et al.*^35^ as structural modification can enhance *T*_lum_ and Δ*T*_sol_. Guo *et al.*^28^ fabricated flexible VO_2_-based thermochromic films by dispersing the VO_2_ powders in a polymer matrix. The *T*_lum_ and Δ*T*_sol_ of these flexible films reached 54.26% and 12.34%, respectively. Cao *et al.*^29^ used the freeze-drying method to prepare VO_2_ film with nanoporous structure, and the film showed the best *T*_lum_ = 50% and Δ*T*_sol_ = 14.7%. Therefore, structurally modified VO_2_ nanoparticles distributed in another dielectric matrix can offer high value of *T*_lum_ and Δ*T*_sol._

Inspired by this concept, 3D architecture with mesopores are introduced in the monoclinic M-phase VO_2_ films. Liu *et al.* fabricated ordered M-phase VO_2_ honeycomb structures with a complex hierarchy by a template-free surface patterning method.^2^ The ordered M-phase VO_2_ honeycomb structure film exhibited a high *T*_lum_ of 95.4%. However, the solar energy modulation ability was only 5.5%. Ke *et al.*^34^ reported 2D patterned M-phase VO_2_ nanonet films with controlled periodicity. The film exhibited a high Δ*T*_sol_ of 13.2%. However, the value of *T*_lum_ was only 46.1%. In this regard, it is a great challenge to introduce surface modification with a desirable architecture in VO_2_ film to obtain excellent performance with balanced *T*_lum_ and Δ*T*_sol_. Most reported works of literature are focused either on surface modification^29,30^ or designing 2D nanoarchitectures.^7,34^ However, the effect of 3D mesoporous structures assembled by VO_2_ nanoflakes based thermochromic films has not been reported so far. Therefore, it is of great importance to investigate the effect of microstructure on 3D mesoporous structure assembled by monoclinic M-phase VO_2_ nanoflakes on the thermochromic properties of VO_2_ films.

Herein, we have prepared 3D mesoporous structures assembled by monoclinic M-phase VO_2_ nanoflakes using a facile hydrothermal method followed by calcination in the presence of *Ensete ventricosum* fiber (EF) as a template. The crystal structure, morphology, shape, composition and pore size distribution of the synthesised materials were investigated by powder XRD, SEM/EDS, TEM, XPS and BET. The film prepared from the synthesised materials were investigated for thermochromic smart window applications.

## 2. Experiments and characterization

### 2.1. Preparation of 3D mesoporous structures assembled by monoclinic M-phase VO_2_ nanoflakes

All reagents were of the analytical grade and were used without further purification. In a typical synthesis, 0.63 g (0.0034 mol) of vanadium pentoxide (V_2_O_5_) and 1.2859 g (0.0102 mol) of oxalic acid (H_2_C_2_O_4_·2H_2_O) were dissolved in 30 ml of deionized water. The suspension was stirred constantly in a water bath kettle at 80 °C until the color of the solution changed to blue. Then 30 ml of ethylene glycol was added to increase the viscosity of the solution. As the acidic solution degrades EF, ammonia was added to adjust the pH to ∼7. The solution and 0.5 g of EF were transferred to a 100 ml Teflon-lined stainless-steel autoclave. The autoclave was maintained at a temperature of 180 °C for 20 h and then cooled to room temperature naturally. The black product with EF was separated and washed with water and ethanol, and then dried in air at 80 °C for 24 h. To remove the template and to obtain the 3D porous structure, the product was calcined at 450 °C for 1 h at a heating rate of 5 °C min^−1^. During the calcination, VO_2_ nanoflakes were oxidized to yellow 3D mesoporous structure assembled by V_2_O_5_ nanoflakes. Finally, the obtained 0.4 g 3D mesoporous structure assembled by V_2_O_5_ nanoflakes and 0.2 g NH_4_HCO_4_ were placed in a vacuum tube furnace for annealing at 550 °C for 30 min. Ammonia gas produced from the decomposition of NH_4_HCO_4_ reduced the 3D mesoporous structure assembled by V_2_O_5_ nanoflakes to 3D mesoporous structure assembled by monoclinic M-phase VO_2_ nanoflakes. For comparison, monoclinic M-phase VO_2_ was also synthesized under similar conditions without EF S1 (ESI).†

### 2.2. Preparation of thermochromic films

The obtained 0.5 g 3D mesoporous structure assembled by, monoclinic M-phase VO_2_ nanoflakes were dispersed in ethanol that contained an appropriate amount of PVP (0.25 g) for subsequent ball milling and sonication to ensure well mixing. After centrifugation, this suspension was then uniformly cast onto a fused silica glass substrate (25 mm × 25 mm × 1 mm) by spin-coating at the speed of 500 rpm for 15 s and then 1500 rpm for 30 s. Finally, the ethanol was removed by drying at 80 °C for 5 min to get the film of 3D mesoporous structure assembled by M-phase VO_2_ nanoflakes based composite.

### 2.3. Characterizations

X-ray diffraction (XRD, D8DISCOVER, Cu Kα1 = 0.154056 nm as the source of radiation, output power 3 kW) was employed to determine the crystal phase of obtained samples. A field emission scanning electron microscopy (FE-SEM, JSM-7500F, JEOL, Japan) and a Hitachi S4800 electron microscope operating at 15 kV were employed to observe the morphology of the nanoflakes and films. Transition electron microscopy (TEM) images were recorded with a (TEM, FEG, Talos F200S, Thermo Fisher, USA) microscope operated at 200 kV. The BET analyzer (ASAP 2020) was used to measure the pore size distribution and the specific surface area with nitrogen as an adsorbate, and the pore size distribution was calculated by the Brunauer–Emmett–Teller (BET) method. The compositions of the powder nanoflakes were detected by X-ray photoelectron spectroscopy (XPS, ESCALAB 250Xi). The phase transition properties were detected by differential scanning calorimeter (DSC, DSC8500, American PE). The thermochromic performance of the films was measured from 300 to 2500 nm by UV-vis-NIR spectrophotometer (UV-3600) equipped with temperature controlling unit at 20 °C and 90 °C, respectively. The integral luminous transmittance (*T*_lum_, 380–780 nm) and solar transmittance (*T*_sol_, 300–2500 nm) were calculated based on the measured spectra using the following expression:^40^*T*_lum/sol_ = ∫*φ*_lum/sol_(*λ*)*T*(*λ*)d*λ*/∫*φ*_lum/sol_(*λ*)d*λ*where *T*(*λ*) denotes the transmittance at the wavelength *λ*, *φ*_lum_(*λ*) is the standard luminous efficiency function for the photopic vision of human eyes, *φ*_sol_(*λ*) is the solar spectral irradiance for the air mass 1.5 corresponding to the sun standing 37° above the horizon. The solar energy modulation ability (Δ*T*_sol_) is defined as: Δ*T*_sol_ = *T*_sol_ (20 °C) − *T*_sol_ (90 °C).

## 3. Results and discussion

### 3.1. Structure and composition of 3D mesoporous structure assembled by monoclinic M-phase VO_2_ nanoflakes

**Scheme 1:**
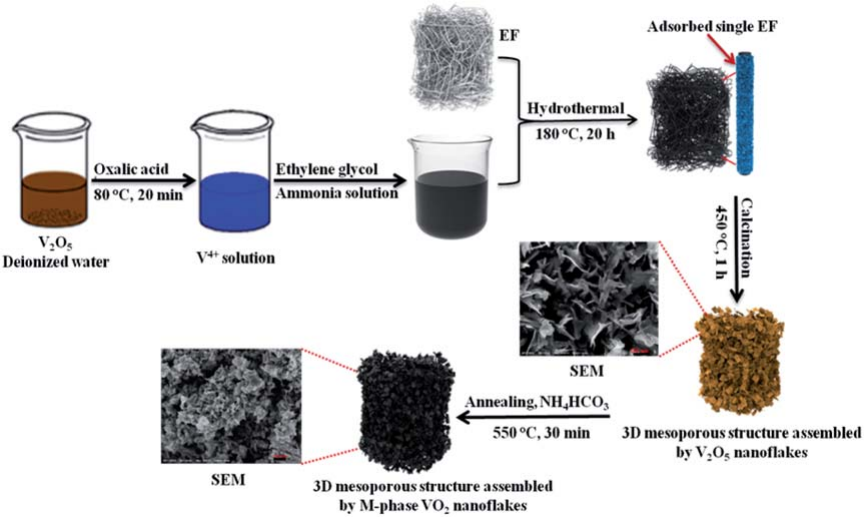
Schematic illustration of the whole process for synthesis of the 3D mesoporous structure assembled by monoclinic M-phase VO_2_ nanoflakes.

Scheme 1 shows the synthesis root and surface morphology of 3D mesoporous structure assembled by monoclinic M-phase VO_2_ nanoflakes. The well-mixed precursor solution was added into Teflon-lined stainless-steel autoclave containing EF for the hydrothermal process for adsorption. The negative surface charge from carboxyl (COO^−^) and other hydrophilic functional groups on the fiber surface facilitate the favorable adsorption of the vanadyl complex.^41^ EF can greatly increase solid–liquid interface so that the nucleation occurs easily on the surface^42^ and then grew into nanoflakes layer by layer interaction with keeping a similar morphology to that of raw EF. In the second step, 3D mesoporous structure assembled by V_2_O_5_ nanoflakes were produced after calcination of the adsorbed fiber at 450 °C in the presence of air to remove the EF template, the results are described in detail in Fig. S2 (ESI).† In this step, VO_2_ nanoflakes were converted into a 3D mesoporous structure assembled by V_2_O_5_ nanoflakes because of the oxidation reaction. The 3D structure and pore formation are directed by EF during the calcination process. Lastly, post-annealing in reducing ammonia atmosphere produced from the decomposition of NH_4_HCO_4_ yields a 3D mesoporous structure assembled by M-phase VO_2_ nanoflakes.

To investigate the effect of the hydrothermal temperature on the structure and pore size, M-phase VO_2_ nanoflakes were synthesised at 160 °C, 180 °C and 200 °C. However, at 160 °C, the experiment failed. The phenomena suggest that temperature is low to facilitate the adsorption and subsequent growth of the nucleus in the surface of EF. The hydrothermal temperature of 200 °C was effective but relatively high energy cost. The obtained result for 200 °C is presented in Fig. S2 (ESI).† As a result, 180 °C is found to be optimum to carry out the experiment.

**Fig. 1:**
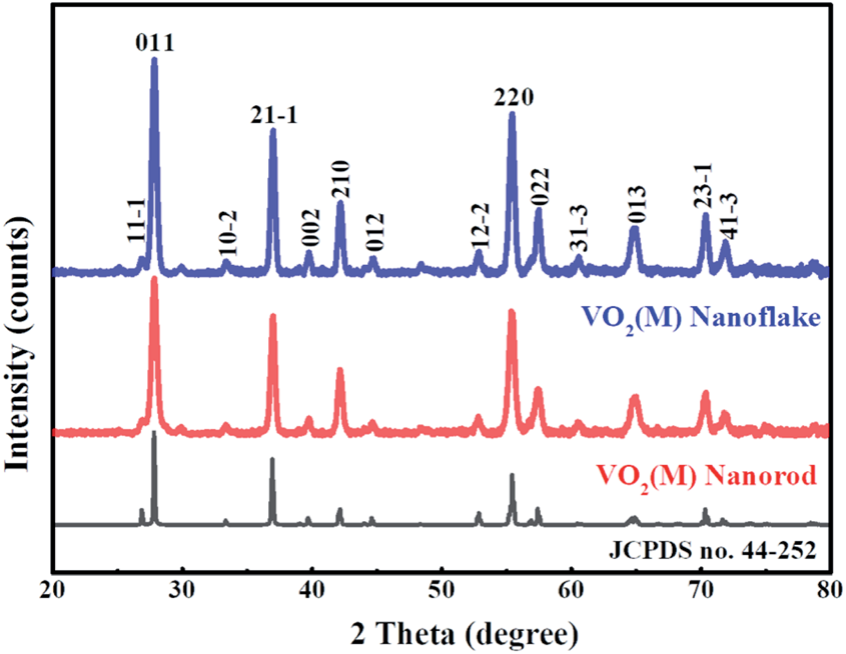
XRD patterns of 3D mesoporous structure assembled by monoclinic M-phase VO_2_ nanoflakes and monoclinic M-phase VO_2_ nanorods (initially prepared at the hydrothermal reaction temperature of 180 °C) obtained by annealing V_2_O_5_ at 550 °C for 30 min in ammonia atmosphere and VO_2_(B) in vacuum, respectively.

The crystal structure and phase composition of samples were measured by XRD (Fig. 1). The diffraction peaks of VO_2_ samples synthesised with and without a template was found to be well fitted to the standard XRD pattern of a monoclinic M-phase VO_2_ phase (space group:*P*2_1_/*c*, (no. 14) JCPDS No. 44-252) and no peaks of any other vanadium oxide phases or impurities are detected. There is no obvious difference in diffraction patterns of the two samples, suggesting that *Ensete ventricosum* fiber (EF) template has no effect on the phase structure of samples. Moreover, the peak intensity of (011) plane of 3D mesoporous structure assembled by M-phase VO_2_ nanoflakes is relatively stronger and narrower than that of the monoclinic M-phase VO_2_ nanorods, indicating a higher crystallinity in the monoclinic M-phase VO_2_ nanoflakes.

**Fig. 2:**
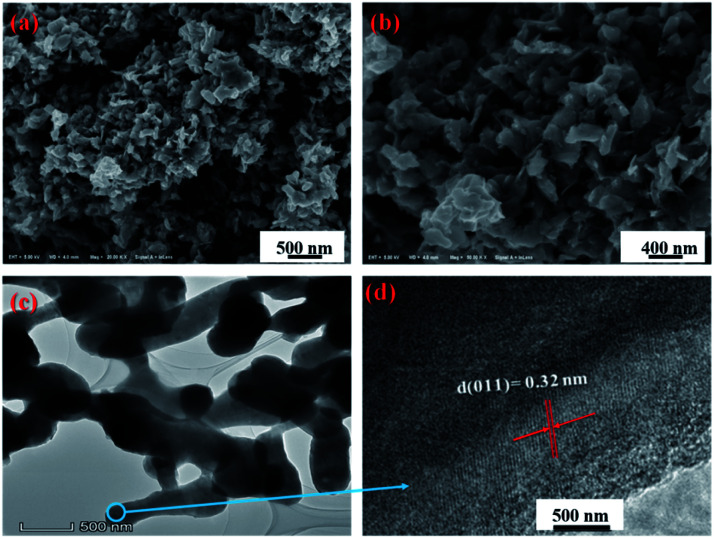
(a) and (b) SEM at different magnification, (c) TEM and (d) HRTEM images of 3D mesoporous structure assembled by monoclinic M-phase VO_2_ nanoflakes.

The morphology of 3D mesoporous structure assembled by monoclinic M-phase VO_2_ were investigated through SEM and TEM. As shown in Fig. 2(a) and (b), a 3D mesoporous structure assembled by monoclinic M-phase VO_2_ exhibits a porous structure composed of uniform nanoflakes with a diameter of ∼90 nm, hundreds of nanometers length and ∼20 nm thickness. Interestingly, the VO_2_ product is 3D mesoporous structure assembled by monoclinic M-phase VO_2_ nanoflakes with many pores. The TEM image of the 3D mesoporous structure assembled by monoclinic M-phase VO_2_ nanoflakes Fig. 2(c), shows the stacks of nanoflakes with many small pores (2–10 nm in the diameter) and some other pores in the range of 10–50 nm in diameter between nanoflakes. This is in good agreement with the morphology observed in the SEM images in Fig. 2(a) and (b). The high-resolution TEM (HRTEM) image Fig. 2(d) taken from the edge of a single nanoflakes Fig. 2(c), shows clear lattice fringes with interplanar *d*-spacing of 0.32 nm, which is corresponding to the (011) lattice plane of monoclinic M-phase VO_2_. This is consistent with the XRD results, indicating high crystallinity for 3D mesoporous structure assembled by monoclinic M-phase VO_2_ nanoflakes.

**Fig. 3:**
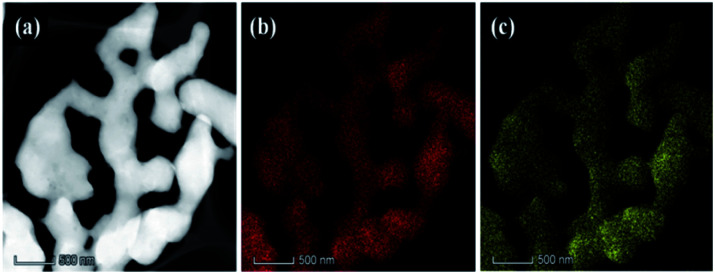
HAADF image (a) and elemental mapping of V (b) and O (c) of obtained 3D mesoporous structure assembled by monoclinic M-phase VO_2_ nanoflakes.

High-angle annular dark-field (HAADF) scanning transmission electron microscopy (STEM) and energy-dispersive spectroscopy (EDS) elemental mapping were used to investigate the chemical analysis of 3D mesoporous structure assembled by monoclinic M-phase VO_2_ nanoflakes (Fig. 3). The HAADF image of obtained 3D mesoporous structure assembled by monoclinic M-phase VO_2_ nanoflakes in Fig. 3(a), shows a 3D uniform stack flakes providing high surface area and mesoporosity. The uniform distribution is also confirmed by EDS recorded from various regions of 3D mesoporous structure assembled by monoclinic M-phase VO_2_ nanoflakes in Fig. 3(b) and (c). The elemental mapping of constituting elements V and O in Fig. 3(b) and (c) demonstrate a well-defined compositional profile of the 3D mesoporous structure assembled by M-phase VO_2_ nanoflakes.

**Fig. 4:**
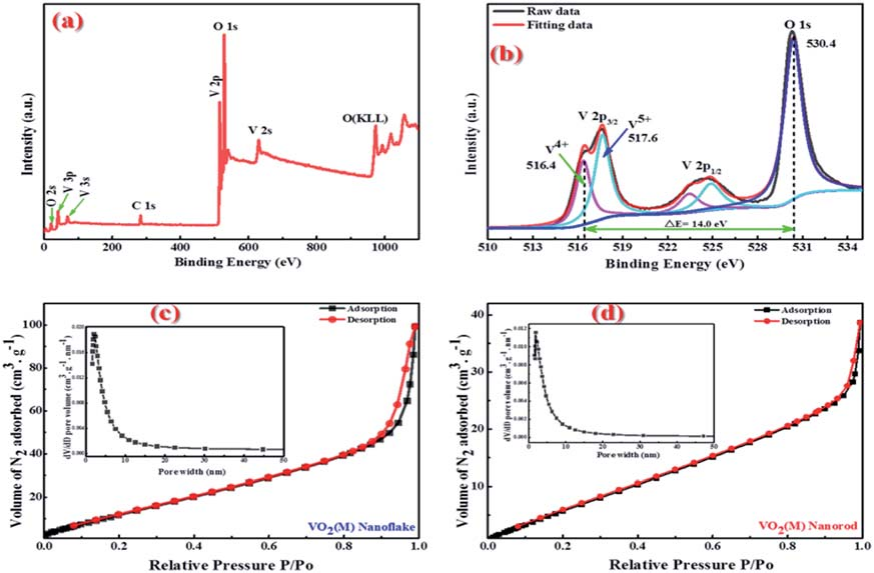
(a) XPS survey spectrum and (b) high resolution XPS spectrum for V2p and O1s of 3D mesoporous monoclinic M-phase VO_2_ nanoflakes, and pore size distribution curves and N2 adsorption–desorption curves of (c) 3D mesoporous monoclinic M-phase VO2 nanoflakes, and (d) monoclinic M-phase VO_2_ nanorods.

The oxidation state of vanadium in the obtained 3D mesoporous structure assembled by monoclinic M-phase VO_2_ nanoflakes was examined by X-ray photoelectron spectroscopy (XPS) and the results are shown in Fig. 4. The fully scanned survey spectra show the presence of only V and O, and no other elements exist in Fig. 4(a). The C1s peak (284.6 eV) in the survey is used as a reference. Fig. 4(b) shows the high-resolution XPS spectra of V2p and O1s peaks as well as their deconvolution which can be fitted by applying a Shirley function with software XPS peak 4.1. The V2p_3/2_ peak can be deconvoluted into two peaks, which correspond to V^5+^ and V^4+^, with the binding energy of 517.6 and 516.4 eV, respectively.^43^ The O1s centered at 530.4 eV can be assigned to the O^2−^ in the V–O.^44^ The presence of V^5+^ ion could be attributed to the partial oxidation of the nanoflakes powder exposed in the air.^43^ Besides, the energy bandgap between V2p_3/2_ and O1s of V^5+^ and V^4+^ are 12.8 and 14.0 eV, respectively, which are in good agreement with reported values of 12.8 and 14.16 eV.^44,45^

BET characterization was carried out to further investigate the porous structure of the synthesized 3D mesoporous structure assembled by monoclinic M-phase VO_2_ nanoflakes. As it is shown in Fig. 4(c), the N_2_ absorption–desorption isotherm belongs to type IV isotherm with H2 hysteresis loop,^46^ which arises from the presence of relatively uniform pore network and ink-bottle shaped pores.^47^ With this isotherm type, capillary condensation at lower adsorbate pressure as well as sharp capillary condensation at higher pressure takes place, indicating that there are abundant mesopores in the 3D mesoporous structure assembled by monoclinic M-phase VO_2_ nanoflakes.^48^ From the pore size distribution curve in the inset of Fig. 4(c), the typical pore size is in the range of 2–10 nm. Furthermore, the total porosity of a material can be categorized into three main groups based on pore size. By IUPAC definition, micropores have pore diameters less than 2 nm, mesopores have pore sizes between 2 and 50 nm, and macropores have pore sizes larger than 50 nm.^49^ The BET surface area and average mean pore size were 42.7 m^2^ g^−1^ and 7.8 nm, respectively. Which confirms the mesoporosity of 3D mesoporous structure assembled by M-phase VO_2_ nanoflakes.

**Fig. 5:**
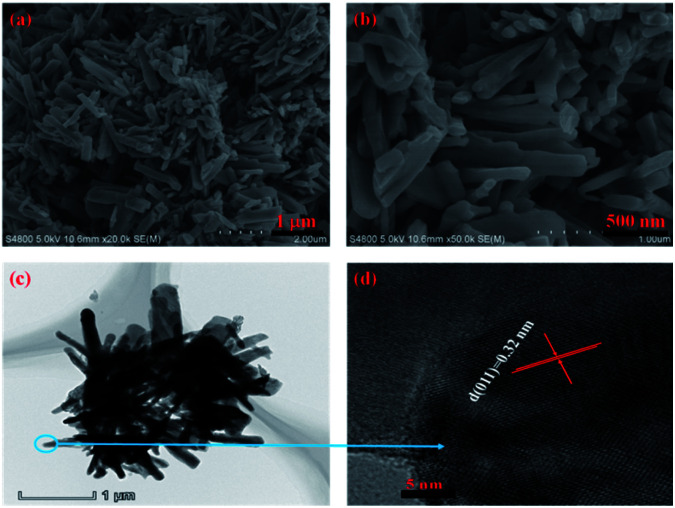
(a) Low- and (b) high-magnification SEM, (c) TEM and (d) HRTEM images of monoclinic M-phase VO_2_ nanorod.

The pore size distribution curves and N_2_ adsorption–desorption curves of M-phase VO_2_ synthesized in the absence of template (monoclinic M-phase VO_2_ nanorod) are shown in Fig. 4(d). It can be seen that the adsorption isotherm displays type IV behavior with H2 hysteresis loop.^46^ From the pore size distribution curve in the inset of Fig. 4(d), the typical pore size is in the range of 2–10 nm. The BET surface area and average mean pore size of nanorods are 19.8 m^2^ g^−1^ and 5.8 nm, respectively. It is believed that mesopores are mainly distributed in M-phase VO_2_ nanorods, which is consistent with TEM results in Fig. 5(c).

The morphology and microstructure of the monoclinic M-phase VO_2_ nanorods were also observed by SEM and TEM, as shown in Fig. 5. The SEM images Fig. 5(a) and (b) reveal that the monoclinic M-phase VO_2_ nanorods are randomly oriented with an average diameter of ∼120 nm. Besides, the nanorods are loosely interconnected with open spaces existing between them, suggesting the formation of small pores. The TEM image Fig. 5(c) unambiguously reveals that the nanorods are interconnected with each other to form their inner space, which is in good agreement with the SEM observations. Fig. 5(d) shows the HRTEM image of monoclinic M-phase VO_2_ nanorods taken from the edge of a single nanorod in Fig. 5(c), the d-spacing of lattice fringe is 0.32 nm, which match well with the (011) lattice plane of monoclinic M-phase VO_2_. This is consistent with the XRD results in Fig. 1.

### 3.2. The thermochromic characteristics of the films

**Scheme 2:**
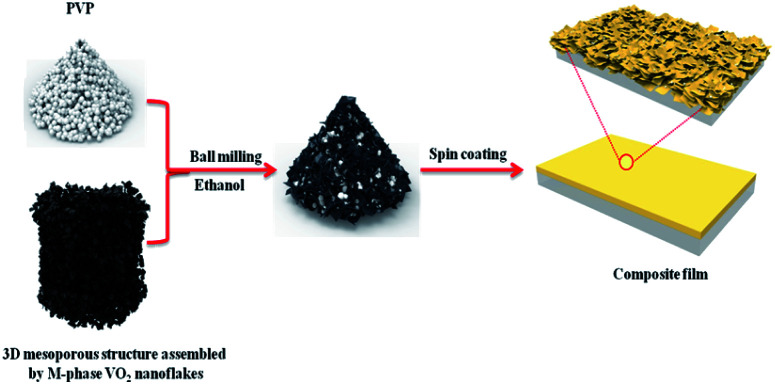
Schematic illustration of the process used for preparation of 3D mesoporous structure assembled by monoclinic M-phase VO_2_ nanoflakes based composite film.

**Fig. 6:**
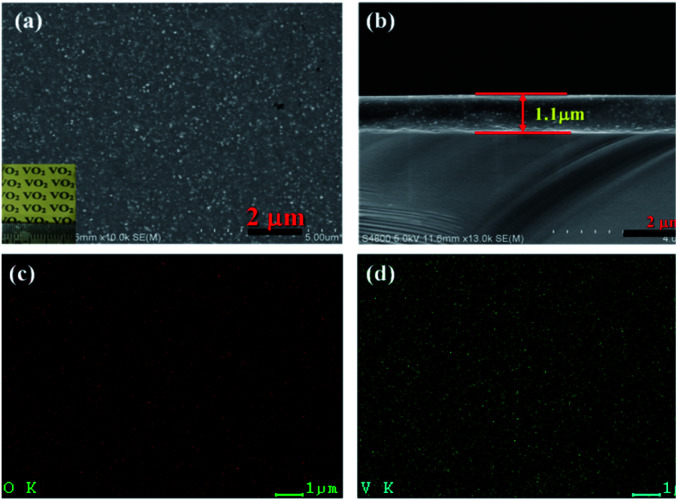
(a) The surface (the inset shows the photograph of film on the glass), (b) the cross-section SEM images, (c) O element distribution and (d) V element distribution of the 3D mesoporous structure assembled by monoclinic M-phase VO_2_ nanoflakes based film.

To investigate the thermochromic properties of thin films, the 3D mesoporous structure assembled by monoclinic M-phase VO_2_ nanoflakes were dispersed in ethanol that contained PVP and the suspension was uniformly cast onto a float glass substrate by spin-coating as shown in Scheme 2. It can be seen from Fig. 6(a), the films are transparent, homogeneous and light brownish-yellow in color. The thickness of the film is about 1.1 μm Fig. 6(b), which is smaller than that of reported composite films,^1,26^ and possesses a high visible light transmittance. To enhance the thermochromic property of the thin film, uniform dispersion of the particles in to the deposited film have crucial effect.^1^ Therefore, the uniform distribution of V and O in the 3D mesoporous structure assembled by M-phase VO_2_ nanoflakes films have been observed by EDS elemental mapping Fig. 6(c) and (d). The figures show the V and O elements are uniformly distributed in the film without agglomeration, indicating a homogeneous dispersion of 3D mesoporous structure assembled by monoclinic M-phase VO_2_ nanoflakes in the film.

**Fig. 7:**
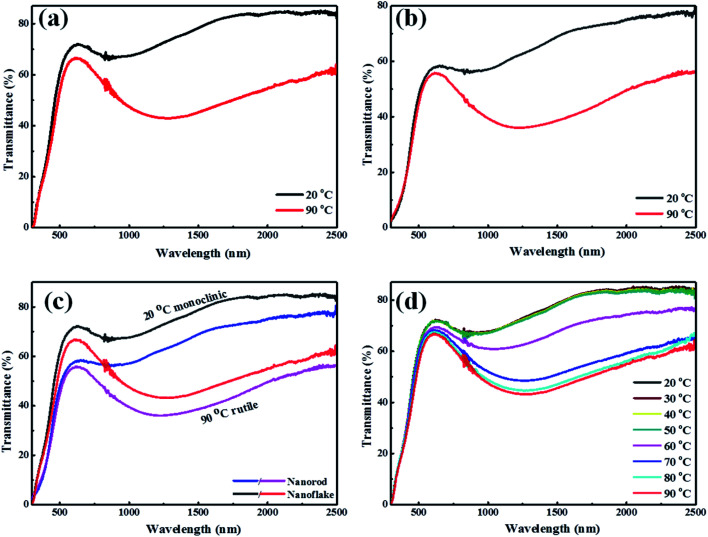
Transmittance spectra of films at 20 °C and 90 °C for (a) 3D mesoporous structure assembled by monoclinic M-phase VO_2_ nanoflakes, (b) M-phase VO_2_ nanorods, (c) comparison of 3D mesoporous structure assembled by monoclinic M-phase VO_2_ nanoflakes and monoclinic M-phase VO_2_ nanorods, and (d) transmittance spectra of 3D mesoporous structure assembled by monoclinic M-phase VO_2_ nanoflakes films at different temperatures.

To further investigate the thermochromic properties of thin films, the UV-vis-NIR transmittance spectra were conducted at different temperatures (Fig. 7). As it is shown in Fig. 7(a), the 3D mesoporous structure assembled by monoclinic M-phase VO_2_ nanoflakes based composite film exhibited excellent optical properties as compared to that of monoclinic M-phase VO_2_ nanorod Fig. 7(b). The calculated optical properties (*T*_lum_ and Δ*T*_sol_) are summarized and compared with previous reports in Table 1.

**Table 1:** Comparison between this work and previously reported high luminous transmittance (*T*_lum_) and solar energy modulation ability (Δ*T*_sol_) of VO_2_ films

System	(*T*_lum_)%	(Δ*T*_sol_)%	Reference
V_*x*_W_1−*x*_O_2_(M)@SiO_2_ NP based films	50.6	14.7	1
VO_2_/NLETS hybrid films	73.4	18.2	15
Zr/W-codoped VO_2_ NP based films	58.4	12.3	20
VO_2_(M) NP based films	62.1	12.4	22
Fine crystalline VO_2_ NP based films	45.6	22.0	26
Flexible VO_2_ NP based films	54.26	12.34	28
Nanoporous M-phase VO_2_ thin films	50.6	14.7	29
Mesoporous SiO_2_/VO_2_ films	80.0	10.2	30
Ordered M-phase VO_2_ honeycomb films	95.4	5.5	2
2D VO_2_/TiO_2_ nanonet films	64.46	11.82	33
2D patterned M-phase VO_2_ nanonet films	46.1	13.2	34
M-phase VO_2_ nanorods films	53.2	10	This work
3D mesoporous structure assembled by monoclinic M-phase VO_2_ nanoflakes film	67.3	12.5	This work

The 3D structure and mesoporosity show great effects on the optical properties. The *T*_lum_ and Δ*T*_sol_ of 3D mesoporous structure assembled by monoclinic M-phase VO_2_ nanoflakes films are 67.3% and 12.5% while that of M-phase VO_2_ nanorods are 53.2% and 10%, respectively. This is because the 3D structure and porosity offer the better dispersion of flakes with PVP during film preparation (Scheme 2) and thereby enhance thermochromic properties. The high luminous transmittance is crucial for smart window^2^ and attributed to the 3D structure and mesoporosity of nanoflakes. The 3D mesoporous structure assembled by monoclinic M-phase VO_2_ nanoflakes also enables the film to contain enough VO_2_ for high solar modulation.^32^ From this, the 3D structure and mesoporosity of nanoflakes play a great role in regulating the visible light transmittance and solar modulation ability. The optical properties of M-phase VO_2_ nanorod film is also good as compared with previous reports. This is because the loosely interconnected nanorods form small pores without the presence of EF. Fig. 7(c) shows the visible transmittance of 3D mesoporous structure assembled by monoclinic M-phase VO_2_ nanoflakes is higher than M-phase VO_2_ nanorods. Fig. 7(d) shows transmittance spectra of 3D mesoporous structure assembled by monoclinic M-phase VO_2_ nanoflakes films at different temperatures in which a critical temperature (*T*_c_) exists between 60 °C and 70 °C. The 3D mesoporous structure assembled by monoclinic M-phase VO_2_ nanoflakes based composite film exhibit much higher balanced *T*_lum_ and Δ*T*_sol_ values than^20,22,28,33^ and slightly lower values than that of.^15^ The 3D mesoporous structure assembled by M-phase VO_2_ nanoflakes based composite film also exhibits higher *T*_lum_ and slightly lower Δ*T*_sol_ values.^1,26,29^ The ordered monoclinic M-phase VO_2_ honeycomb films^2^ exhibited higher *T*_lum_ but Δ*T*_sol_ was too low. In this regards, 3D mesoporous structure assembled by M-phase VO_2_ nanoflakes based composite film exhibit excellent balanced performance between *T*_lum_ and Δ*T*_sol_.

### 3.3. Metal-to-insulator transition properties of 3D mesoporous structure assembled by monoclinic M-phase VO_2_ nanoflakes

**Fig. 8:**
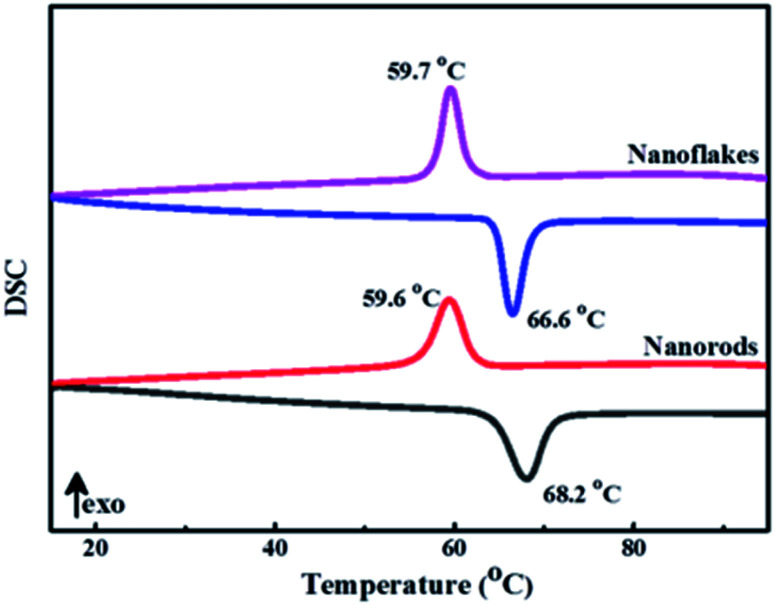
DSC curves of 3D mesoporous structure assembled by monoclinic M-phase VO_2_ nanoflakes and monoclinic M-phase VO_2_ nanorods.

The metal-to-insulator transition properties of 3D mesoporous structure assembled by M-phase VO_2_ nanoflakes and monoclinic M-phase VO_2_ nanorod was determined by differential scanning calorimeter (DSC). As shown in Fig. 8, both DSC curves exhibit a clear endothermic peak and an exothermic peak corresponding to a reversible phase transition between M-phase and R-phase. The endothermic transition temperature (*T*_c,h_) during heating cycles was observed at 66.6 °C and an exothermic transition temperature (*T*_c,c_) during cooling cycles was observed at 59.7 °C for 3D mesoporous structure assembled by monoclinic M-phase VO_2_ nanoflakes. So, the phase transition temperature of 63.15 °C is calculated by *T*_c_ = (*T*_c,h_ + *T*_c,c_)/2,^37^ which is lower than 63.9 °C for M-phase VO_2_ nanorods, ordered honeycomb,^2^ nanorings,^21^ nanobelts^11^ and 68 °C for the bulk. The hysteresis width (Δ*T* = *T*_c,h_ − *T*_c,c_) of 3D mesoporous structure assembled by monoclinic M-phase VO_2_ nanoflakes is 6.9 °C, which is much lower than previously reported values^2,11,19,21,25^ and also lower than that of M-phase VO_2_ nanorod 8.6 °C, but slightly higher than that of nanobelts.^11^ This reduction of *T*_c_ by 3D mesoporous structure assembled by M-phase VO_2_ nanoflakes is due to the uniform dispersion of VO_2_. Table 2 summarizes phase transition temperature for heating and cooling cycles of M-phase VO_2_ with different morphologies. However to get a further reduction of *T*_c_ modification of morphology,^50^ size,^51^ defect,^52^ interfacial effect,^11^ Stress,^52^ and phase purity^53^ have been reported. Particularly doping of metals such as W,^54–57^ Mo,^57,58^ and Nb^57,58^ were reported for the reduction of *T*_c_. Hence, synthesis materials with optimum *T*_lum_, *T*_sol_, and *T*_c_ values for practical thermochromic applications are required.

**Table 2:** Comparison of phase transition temperature for heating and cooling cycles of 3D mesoporous structure assembled by M-phase VO_2_ nanoflakes with previously reported different morphologies

M-phase VO_2_ morphology	*T*_c,h_	*T*_c,c_	*T*_c_	Δ*T*	Reference
Ordered honeycomb	81	59	70	22	2
Star-shaped nanoparticles	68.6	55.2	61.9	13.4	11
Cucumber-like shape	67.5	57.9	62.7	9.6	19
Nanorings	68.75	59.77	64.26	8.98	21
Belt with a rectangular cross-section	66	57	61.5	9	25
Nanobelts	68.9	62.44	65.67	6.46	11
Nanorods	68.2	59.6	63.9	8.6	This work
3D assembled by nanoflakes	66.6	59.7	63.15	6.9	This work

## 4. Conclusion

In conclusion, 3D mesoporous structure assembled by monoclinic M-phase VO_2_ nanoflakes with pore size of about 2–10 nm were synthesized by a facile hydrothermal method at temperature of 180 °C using *Ensete ventricosum* fiber (EF) as a template. The EF facilitates the 3D structure and pore formation in the VO_2_ nanoflakes and thus enhances thermochromic properties. The composite film obtained from 3D mesoporous structure assembled by monoclinic M-phase VO_2_ nanoflakes with PVP exhibited excellent thermochromic properties: a high luminous transmittance (*T*_lum_ = 67.3%) and high solar modulation efficiency (Δ*T*_sol_ = 12.5%), as well as a low phase transition temperature of (*T*_c_ = 63.15 °C) which is lower than bulk (68 °C). In comparison, the M-phase VO_2_ nanorod film exhibits inferior luminous transmittance and solar modulation efficiency as well as a relatively high phase transition temperature. The excellent thermochromic properties of 3D mesoporous structure assembled by M-phase VO_2_ nanoflakes film compared with M-phase VO_2_ nanorods as well as previous reports is to attributed to 3D structure and mesoporosity. Besides, the 3D mesoporous structure assembled by monoclinic M-phase VO_2_ nanoflakes possesses a comparatively lower *T*_c_ and a narrower thermal hysteresis width compared with other monoclinic M-phase VO_2_ materials. Therefore, this green route opens the path for synthesis of 3D structure in VO_2_ and its application in smart windows.

## Conflicts of interest

There are no conflicts to declare.

## Supplementary Material

RA-011-D1RA01558C-s001
