# Supplementary material for: 3D mesoporous structure assembled from monoclinic M-phase VO2 nanoflakes with enhanced thermochromic performance
Source: RSC Adv. 2021 Apr 13;11(22):13556–63. doi: 10.1039/d1ra01558c (PMC8697583; doi:10.1039/d1ra01558c)
Supplement: RA-011-D1RA01558C-s001 [file RA-011-D1RA01558C-s001.pdf]

Electronic Supplementary Material (ESI) for RSC Advances.
This journal is © The Royal Society of Chemistry 2021

Supporting information:

3D mesoporous structure assembled by monoclinic M-phase VO_2 nanoflakes with enhanced thermochromic performance

Liboro Hundito Molloro,[a, b, *] Shouqin Tain,[a] Neway Belachew, [b] Kwadwo Asare Owusu,[c] Xiujian Zhao[a,*]

[a]State Key Laboratory of Silicate Materials for Architectures, Wuhan University of Technology (WUT), No. 122, Luoshi Road, Wuhan 430070, P. R. China

[b]Department of Chemistry, Debre Berhan University, P.O.BOX 445, Debre Berhan, Ethiopia

[c]State Key Laboratory of Advanced Technology for Materials Synthesis and Processing, Wuhan University of Technology (WUT), No. 122, Luoshi Road, Wuhan 430070, P. R. China

*Corresponding author. E-mail address: liborotg@yahoo.com

S1. Synthesis procedure of monoclinic M-phase VO_2 nanorods

In a typical synthesis, 0.63 g (0.0034 mol) of vanadium pentoxide (V_2O_5) and 1.2859 g (0.0102 mol) of oxalic acid ($H_2C_2O_4.2H_2O$) were dissolved in 30 ml of deionized water. The suspension was stirred constantly in a water bath kettle at 80 °C until the color of the solution changed to blue. Then 30 ml of ethylene glycol was added to increase the viscosity of the solution. The solution was transferred to a 100 ml Teflon-lined stainless-steel autoclave. The autoclave was maintained at a temperature of 180 °C for 20 h and then cooled to room temperature naturally. The obtained black product was washed with water and ethanol, and then dried in air at 80 °C for 24 h to obtain monoclinic M-phase VO_2 nanorods.

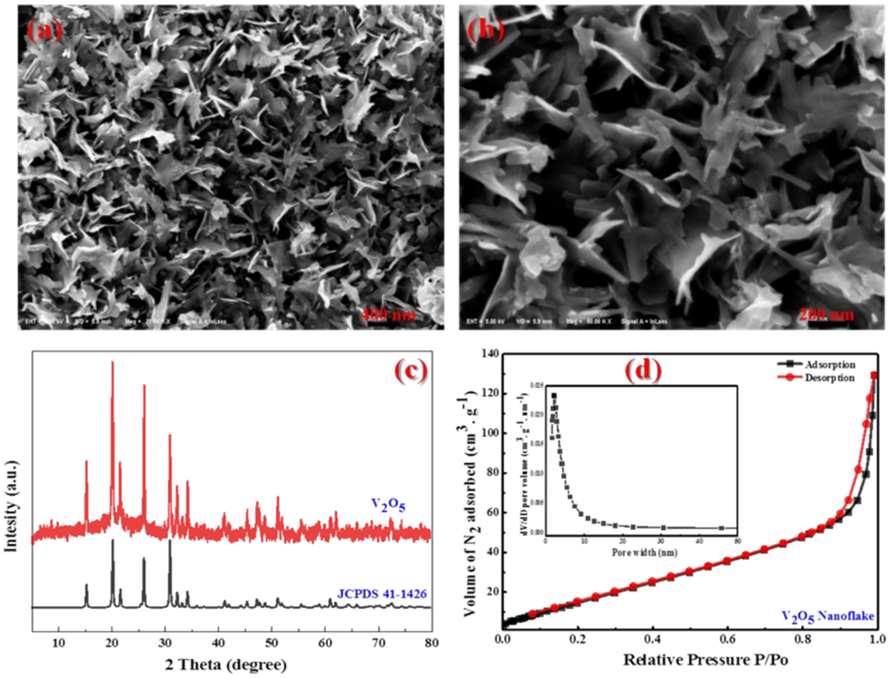

Fig. S1. 3D mesoporous structure assembled by V_2O_5 nanoflakes (a) SEM images low and (b) high magnification, (c) XRD patterns of V_2O_5 (JCPDS: 41-1426) and (d) Pore size distribution curves (the inset)and N_2 adsorption-desorption curves produced after calcinations of the adsorbed fiber at 450 ^{o}C in the presence of air to remove the EF template (initially prepared at the hydrothermal reaction temperature of 180 ^{o} C).

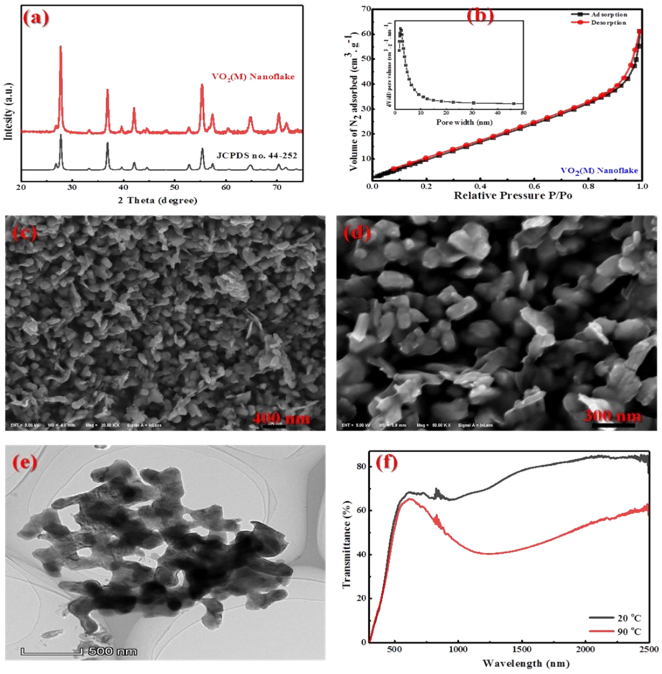

Fig. S2. 3D mesoporous structure assembled by M-phase VO_2 nanoflakes (a) XRD patterns, (b) Pore size distribution curves (the inset) and N_2 adsorption-desorption curves, (c) SEM images low and (d) high magnification, (e) TEM image and (f) Transmittance spectra of films at 20 ^{o}C and 90 ^{o}C (prepared at the hydrothermal reaction temperature of 200 ^{o} C)
